# A61 ELUCIDATING INTESTINAL EPITHELIAL INFLAMMASOME MECHANISMS THAT REPAIR THE MUCOSAL BARRIER DURING ENTERIC INFECTION

**DOI:** 10.1093/jcag/gwad061.061

**Published:** 2024-02-14

**Authors:** A Ranger, S Goyal, S E Girardin

**Affiliations:** Laboratory Medicine and Pathobiology, University of Toronto, Toronto, ON, Canada; Laboratory Medicine and Pathobiology, University of Toronto, Toronto, ON, Canada; Laboratory Medicine and Pathobiology, University of Toronto, Toronto, ON, Canada

## Abstract

**Background:**

To protect the gastrointestinal tract from infection, intestinal epithelial cells (IECs) lining the mucosa possess cytosolic complexes called inflammasomes which detect pathogenic insult and rapidly initiate inflammation to stop infectious spread. Active inflammasomes mediate crucial effector functions such as epithelial cell extrusion, pyroptotic cell death, and the release of interleukin (IL)-18 and prostaglandin E_2_ (PGE_2_). Recent literature has shown that IL-18 and PGE_2_ mediate intestinal repair following epithelial damage from DSS-induced colitis. Exogenous administration of IL-18 in inflammasome deficient mice improves survival and reduces pathology. PGE_2_ activates intestinal cellular reprogramming to drive intestinal wound healing. Whether inflammasome derived IL-18 and PGE_2_ mediate epithelial repair during enteric infection is unclear.

**Aims:**

Using murine small intestinal organoids (mSIOs), we will investigate how IL-18 and PGE_2_ production by intestinal inflammasomes directly impacts IECs at both transcriptional and physiological levels. We hypothesize that inflammasome derived IL-18 and PGE_2_ will act on the intestinal epithelium resulting in transcriptional reprogramming that promotes tissue regeneration and barrier reinforcement.

**Methods:**

WT and *Nlrc4*^*-/-*^ mSIOs were stimulated with flagellin or FlaTox to activate the NAIP-NLRC4 inflammasome. The transcriptomic landscape was assessed with bulk-RNA sequencing and inflammasome activation was assessed by Western blot. IL-18 and PGE_2_ dependent gene targets were identified using *Il-18*^*-/-*^ and indomethacin treated mSIOs by RT-qPCR.

**Results:**

Bulk-RNA sequencing revealed an inflammasome activation dependent transcriptional signature in murine IECs upon NAIP-NLRC4 inflammasome activation. GO term analysis showed these genes to be involved in actin cytoskeleton rearrangement, epidermal growth factor signaling, and regulation of cell-cell adhesion. The expression of PGE_2_ gene targets were significantly reduced by indomethacin treatment while IL-18 gene targets remained unaffected in *Il-18*^*-/-*^ IECs. FlaTox induced the same transcriptional changes but substantially amplified their expression. FlaTox but not flagellin led to a significant drop in *Lgr5* (stem cells) and *Lyz1* (Paneth cell) expression and an increase in *Ly6a,* a marker of fetal-like stem cells.

**Conclusions:**

This data demonstrates that inflammasome signaling causes transcriptional reprogramming of IECs that activates pathways involved in maintaining epithelial barrier integrity. While IL-18 had no effects on gene expression at 4 hours, PGE_2_ paracrine signaling contributed to the expression of wound healing genes. Stronger inflammasome mediated damage with FlaTox amplified the repair response at the transcript level and we observed transient reprogramming of IECs into a fetal-like state suggesting active tissue regeneration.

**Funding Agencies:**

CCC, CIHREPIC: Emerging & Pandemic Infections Consortium

